# Diagnostic performance of salivary urea nitrogen dipstick to detect and monitor acute kidney disease in patients with malaria

**DOI:** 10.1186/s12936-018-2627-4

**Published:** 2018-12-18

**Authors:** Viviane Calice-Silva, Euclides Sacomboio, Jochen G. Raimann, Rhys Evans, Cruz dos Santos Sebastião, Adelino Tchilanda Tchivango, Peter Kotanko, Nathan Levin, Roberto Pecoits-Filho

**Affiliations:** 1 School of Medicine, Pontifícia Universidade Católica do Paraná, Curitiba, Brazil; 2 Pro-Rim Foundation, Joinville, Brazil; 3 Higher Institute of Health Sciences/Agostinho Neto University, Luanda, Angola; 4 Research Division, Renal Research Institute, New York, USA; 5 Nephrology Department, Queen Elizabeth Central Hospital (QECH), Blantyre, Malawi; 6 University College London Centre for Nephrology, London, UK; 7 Icahn School of Medicine at Mount Sinai, New York, USA

**Keywords:** Salivary urea nitrogen, Diagnostic tools, Blood urea nitrogen, Acute kidney disease, Acute kidney injury, Malaria

## Abstract

**Background:**

Acute kidney injury (AKI) is a common complication of malaria. In low resource settings, a lack of diagnostic tools and delayed treatment of malaria associated AKI lead to significant morbidity and mortality. The aim of this study was to assess the diagnostic performance of salivary urea nitrogen (SUN) dipstick to detect and monitor kidney disease [KD = AKI or acute kidney disease (AKD) without AKI] in malaria patients in Angola.

**Methods:**

Patients 11–50 years old admitted with malaria at the Josina Machel (Maria-Pia) Hospital, Luanda, Angola, between 2nd March and 10th May 2016 were enrolled in this study. All participants had serum creatinine (sCr), blood urea nitrogen (BUN) and SUN dipstick tested at the time of recruitment and daily for up to 4 days. AKD without AKI refers to acute renal impairment which do not fulfilled the main criteria for AKI (increases in the baseline serum creatinine and/or decreases in urine output) according defined by the kidney disease improving global outcomes (KDIGO) guideline.

**Results:**

Eight-six patients were admitted with malaria diagnosis (mean age 21.5 ± 9.4 years, 71% male) and 27 (32%) were diagnosed with KD. The mean (± SD) sCr and BUN of the KD group at admission (day 0) were 5.38 (± 5.42) and 99.4 (± 61.9) mg/dL, respectively. Three (3.5%) patients underwent haemodialysis and eight (9.3%) died within the first 4 days of hospital admission [5 (62.5%) with KD; 3 (37.5%) without kidney disease; p = 0.047]. The SUN threshold for KD diagnosis was tested pad #5 (SUN > 54 mg/dL). At this threshold, the SUN dipstick had a sensitivity of 67% and specificity of 98% to diagnose KD. The area under the receiver operating characteristics curve (ROC) for KD diagnosis on admission was 0.88 (95% CI 0.79–0.96). The SUN dipstick was most accurate at higher levels of BUN.

**Conclusion:**

The SUN dipstick had reasonable sensitivity and excellent specificity when used to diagnose KD in a cohort of patients with malaria in a resource-limited setting. Given the severity of presenting illness and kidney injury, the SUN dipstick diagnostic threshold was high (test pad #5). SUN may be used to detect AKI in patients with malaria in low resources settings, thus facilitating earlier access to adequate treatment, which may improve survival.

**Electronic supplementary material:**

The online version of this article (10.1186/s12936-018-2627-4) contains supplementary material, which is available to authorized users.

## Background

Despite control efforts, malaria remains one of the most common infectious diseases in more than 90 countries and territories worldwide (300–500 million cases per year estimated by the World Health Organization [1]). Most deaths due to the disease occur in children below the age of 5 years old in Africa, particularly in rural areas with limited or no access to medical care [2–4]. Governments of endemic countries contributed 31% of total funding (US$ 800 million) and the majority (74%) of investments were spent in the African Region followed by the regions of South-East Asia (7%) [4]. However, all these investments are not enough in some regions where the lack of diagnostic tools is an important problem to detect and treat the disease and its complications.

One of the most serious complications of malaria is the acute impairment of renal function, mainly in patients with falciparum and vivax malaria [5, 6]. The pathogenesis of malaria associated AKI is unclear, however, several factors have been suggested: tubular obstruction by parasitized erythrocytes; an excessive host immune response with reactive oxygen and nitrogen species production; interstitial inflammation and deposition of immune complexes; hypovolaemia and renal microcirculation disorders leading to acute tubular injury; and peri-infectious proliferative glomerular changes with thickening of the basement membrane [7, 8].

The presence of AKI is a strong predictor of mortality in malaria [9, 10]. In low resource settings, as in most African countries with high malaria incidence, AKI increases morbidity and mortality particularly due to lack of early diagnosis and treatment. Laboratory staff and resources are scarce and expensive in these areas where malaria is first diagnosed and managed [11–13]. The measurement of newer biomarkers such as cystatin C, kidney injury molecule-1 (KIM-1) and neutrophil gelatinase-associated lipocalin (NGAL) to diagnose AKI is unavailable in developing countries, and even tests that are routine in higher income countries, such as serum creatinine (sCr), are often unavailable [14, 15]. Simple bedside tools to detect renal impairment can, therefore, facilitate early diagnosis and adequate treatment, and thus may improve patient outcomes [16].

While several point-of-care (POC) testing methods and devices such as those developed to measure serum creatinine at the bedsite (e.g. iSTAT^®^, Stat senso^®^, Piccolo xpress^®^ and others), are available to assess kidney function, notably at considerably lower costs compared to lab facilities, these tests are still too expensive for widespread use [16]. In addition, most tests are based on enzymatic methods to measure creatinine and may thus require refrigeration of test strips pads, rendering their use challenging in low resource settings with hot and humid climates [16]. Previous publications have reported the diagnostic performance of a salivary urea nitrogen (SUN) dipstick to detect acute and chronic kidney disease in both developed and developing countries in patients with different chronic kidney disease (CKD) stages, including patients undergoing dialysis, and also AKI patients with various aetiologies [17–21].

Considering malaria is a community-acquired disease, highly prevalent in many low-resource regions worldwide, the use of POC testing at the primary care facilities, in small villages, would increase the chances of acute kidney disease detection and referral to specialized care improving the chances to receive an adequated treatment to that serious complication, consequently increasing survival among this group of patients. The aim of this study was to evaluate the diagnostic performance of the salivary urea nitrogen (SUN) dipstick to detect and follow acute kidney disease in malaria patients in Angola.

## Methods

### Study design

A longitudinal prospective observational study was conducted after been approved by the Research Ethics Committee from the Instituto Superior de Ciências de Saúde from Universidade Agostinho Neto, Luanda, Angola (no. 664/GD/ISCISA/UAN/2015), with the Hospital Josina Machel (Maria Pia) manager authorization (no. 23/DPC/HJM/2016). The Josina Machel Hospital is a tertiary hospital located in Luanda, Angola, reference for the treatment of chronic diseases and also for complicated malaria in the Luanda’s region. Patients between 11 and 50 years old (small kids up to 12, teenagers up to 18 and adults ≥ 18 years old), hospitalized with malaria at the Josina Machel (Maria Pia) Hospital in Luanda, Angola, between 2nd March and 10th May 2016 were approached for consent. In all patients who agreed to participate and signed an informed consent form, blood was drawn at admission to measure sCr and BUN. In addition, the patients provided saliva samples for SUN measurements using a dipstick. Presence of AKI was defined according to the KDIGO criteria, based on sCr levels. Patients enrolled were followed for up to 4 days while hospitalized. Blood and salivary samples were collected daily for the measurement of sCr, BUN and SUN. Patients unable to give the inform consent, to provide saliva samples and those with known CKD or with other chronic diseases which could potentially cause CKD prior to the hospitalization, such as hypertension and diabetes, were excluded from the study to minimize the odds of recruiting subjects with preexisting kidney impairment.

### SUN measurement

**Fig. 1:**
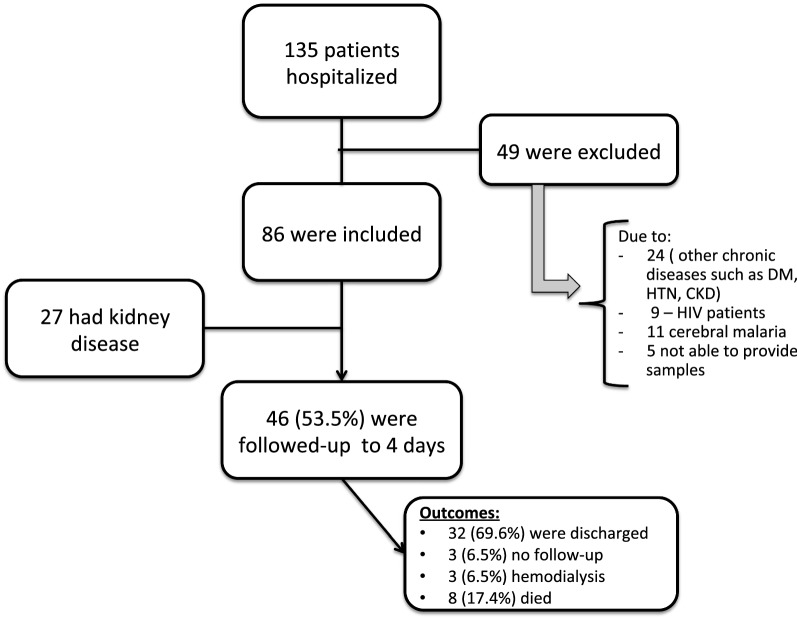
Flowchart—study population description

Unstimulated saliva and blood were collected concurrently when blood was drawn. Subjects were asked to refrain from drinking and eating for at least 15 min prior to saliva collection. Saliva was sampled in a plastic cup and approximately 50 μL of its liquid fraction were used to moisten the colorimetric SUN dipstick. After 1 min the colour of the test pad was compared to six standardized colour fields indicating SUN concentrations of 5–14 (colour pad #1), 15–24 (#2), 25–34 (#3), 35–54 (#4), 55–74 (#5), and ≥ 75 (#6) mg/dL, respectively (Fig. 1 and Additional file 1: Figure S1).

### Blood sample analysis

For BUN and sCr tests, 3–5 mL of blood were collected from a peripheral vein and processed in automated devices (Vital Scientific Flexor E180 and Flexor E450) in the central laboratory of the Josina Machel Hospital.

### Malaria diagnosis

Diagnosis of malaria was made by either thick blood smear analysis or rapid malaria antigen test (SD-Bioline Malaria AG Pf/PAN). The tests were performed by Josina Machel hospital professionals, who subsequently also confirmed the diagnosis by direct parasite visualization using Giemsa-staining of a thick blood drop. All these tests were done on the day of patient’s inclusion into the study [22, 23].

### Acute renal disease classification

AKI and AKD without AKI were classified according to KDIGO criteria, by the serum creatinine criteria only, because urine output of these patients was not evaluated during hospitalization period, thus not assessed in the research population as well [24]. AKD without AKI refers to those patients who have serum creatinine elevated at the admission and does not fulfill the criteria to be classified into one of the AKI stages by KDIGO neither trough serum creatinine nor trough urine output variation, it was considered acute renal impairment known or presumed to be of < 3 months duration but not fulfilling the main criteria for AKI according KDIGO guideline definition. In children, an assumed baseline eGFR (according to bedside Schwartz equation) of 100 mL/min/1.73 m^2^ was used [25]. AKI and AKD without AKI were combined under the kidney disease (KD) group for the statistical analyses and results report.

### Statistical analysis

Continuous data are summarized as mean ± standard deviation if normally distributed, otherwise as median and interquartile range. Differences between BUN and SUN (transformed to a continuous variable by choosing the midpoint of each test pad) are depicted with their 95% confidence intervals. A modified Bland–Altman plot with BUN as the reference was also constructed.

Agreement between SUN and BUN over the entire period assessed using linear mixed effects models with the study subject as the random intercept and the measurement day as the random slope. Diagnostic performance of SUN and BUN to detect KD were analysed by computing the sensitivity and specificity with KD as a binary outcome, and the area under the receiver operating characteristics curve at each of the 4 observation days following the approach described previously [18]. Youden index was computed as follows: true positive (TP) was a patient with KD (as a dichotomous outcome) and a positive test result. A patient was considered as true negative (TN) if the SUN level was below the determined threshold in the absence of KD. False positive (FP) and false negative (FN) results were defined accordingly. Sensitivity was computed as TP/(TP + FN), and specificity as TN/(TN + FP). Optimal diagnostic thresholds were determined based on the maximum of the Youden’s index (Youden’s index = sensitivity + specificity − 1).

A two-sided p-value < 0.05 was considered as statistically significant. Analyses were done with R 3.2.1 (codename ‘World-Famous Astronaut’; R Foundation for Statistical Computing; Vienna, Austria) additionally using the packages *plyr*, *sandwich*, *nlme*, *multcomp*, *pROC* and *ggplot2* [26].

## Results

### Cohort description

During the study period 135 patients were admitted at the hospital with malaria, and, 86 (63.7%) were included in the study (Fig. 1). Mean age was 21.5 (± 9.4) years, 61 (71%) were male and 27 (32%) had KD [AKI = 15 (55.6%) + AKD without AKI = 12 (44.4%)]. The KDIGO stage most prevalente was the stage 1 with 8 (29.7%) patients in this stage, other 4 (14.8%) patients were at the stage 2 and only 3 (11.1%) at stage 3. sCr and BUN of the KD group at admission (day 1) were 5.38 ± 5.42 and 99.4 ± 61.9 mg/dL, respectively. Three patients (3.5%) received haemodialysis and eight (9.3%) patients died during the 4-days follow-up. Of those patients who died, 5/8 (62.5%) had KD, compared to 3/8 (37.5%) with no KD (p = 0.047). Sixty percent of patients were undergoing malaria treatment prior to the study recruitment and hospital admission (self-medication or first care in health care centres) (Table 1).

**Table 1:** Descriptive analyses final cohort (N = 86)

Variable	Value
Age (years)	21.5 ± 9.5
Male (N; %)	61 (71%)
Presence of kidney disease (N; %)	27 (32%)
BUN at day 1 (mg/dL)	39.5 ± 14.9
sCr at day 1 (mg/dL)	5.38 ± 5.42
KDIGO—AKI stage 1 (N; %)	8 (29.7%)
KDIGO—AKI stage 2 (N; %)	4 (14.8%)
KDIGO—AKI stage 3 (N; %)	3 (11.1%)
AKD no AKI (N; %)	12 (44.4%)

KDIGO classification: (a) AKI stage 1: variation of 1.5–1.9 times baseline serum creatinine OR ≥ 0.3 mg/dL (≥ 26.5 mmol/L) increase; (b) AKI stage 2: variation of 2.0–2.9 times baseline serum creatinine; (c) AKI stage 3: variation of 3.0 times baseline serum creatinine OR increase in serum creatinine to ≥ 4.0 mg/dL (≥ 353.6 mmol/L) OR initiation of renal replacement therapy OR, in patients < 18 years, decrease in eGFR to < 35 mL/min per 1.73 m^2^

*BUN* blood urea nitrogen, *sCR* serum creatinine

### SUN and BUN measurements

**Fig. 2:**
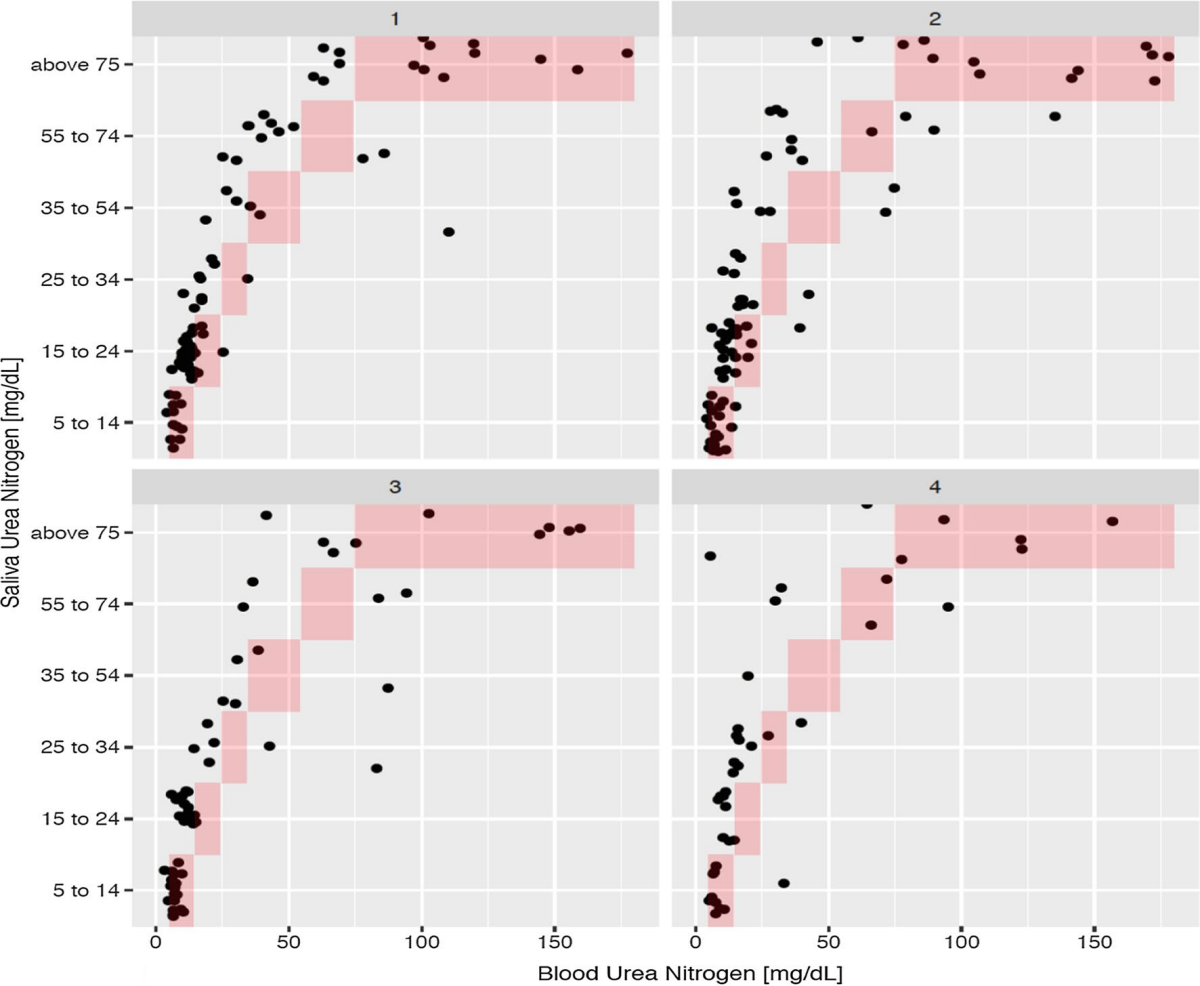
Scatter plot showing the relationship between salivary urea nitrogen and blood urea nitrogen on days 1–4. The red areas represent the ranges of the semiquantitative saliva urea nitrogen dipsticks

**Fig. 3:**
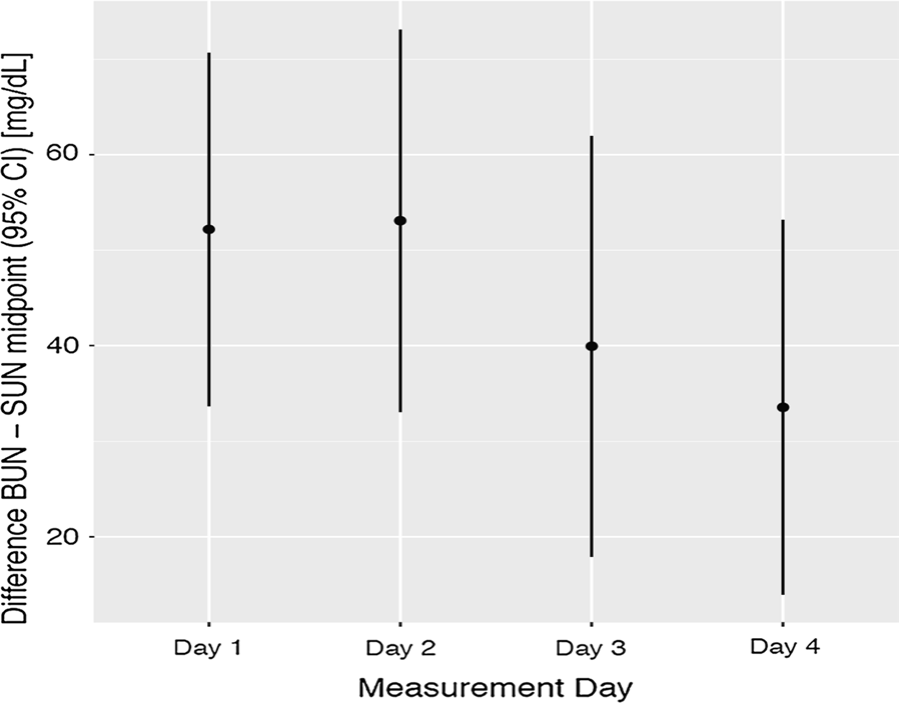
Bland Altman plot showing the mean differences and 95% confidence intervals between BUN and SUN. SUN was transformed to a continuous variable by choosing the midpoint for each test pad range

While BUN was overestimated by SUN in this population (Table 2), there was a good directional agreement along the follow-up day’s period (day 1 to day 4) (Fig. 2). Plotting the differences (BUN–SUN midpoint) as a function of BUN a modified Bland–Altman plot showed a consistent non-significant bias with a proportional error (Fig. 3). The linear mixed effects model with SUN as the dependent variable and BUN as a fixed effect with random intercept (individual patient) and random slope (individual observation day) showed a significant continuous association between SUN and BUN (0.40 mg/dL SUN per mg/dL BUN; p < 0.001).

**Table 2:**
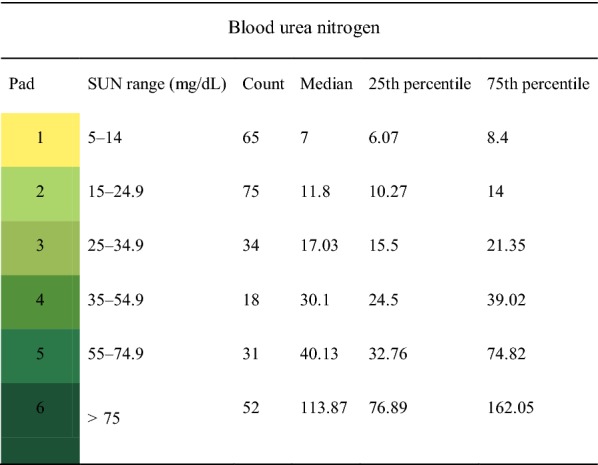
Median and 25th and 75th blood urea nitrogen percentiles corresponding to SUN test strip pads

The colour scheme in the left column indicates the test actual pad colours

*SUN* salivary urea nitrogen

#### Diagnostic performance of SUN and BUN for AKI and AKD without AKI

**Fig. 4:**
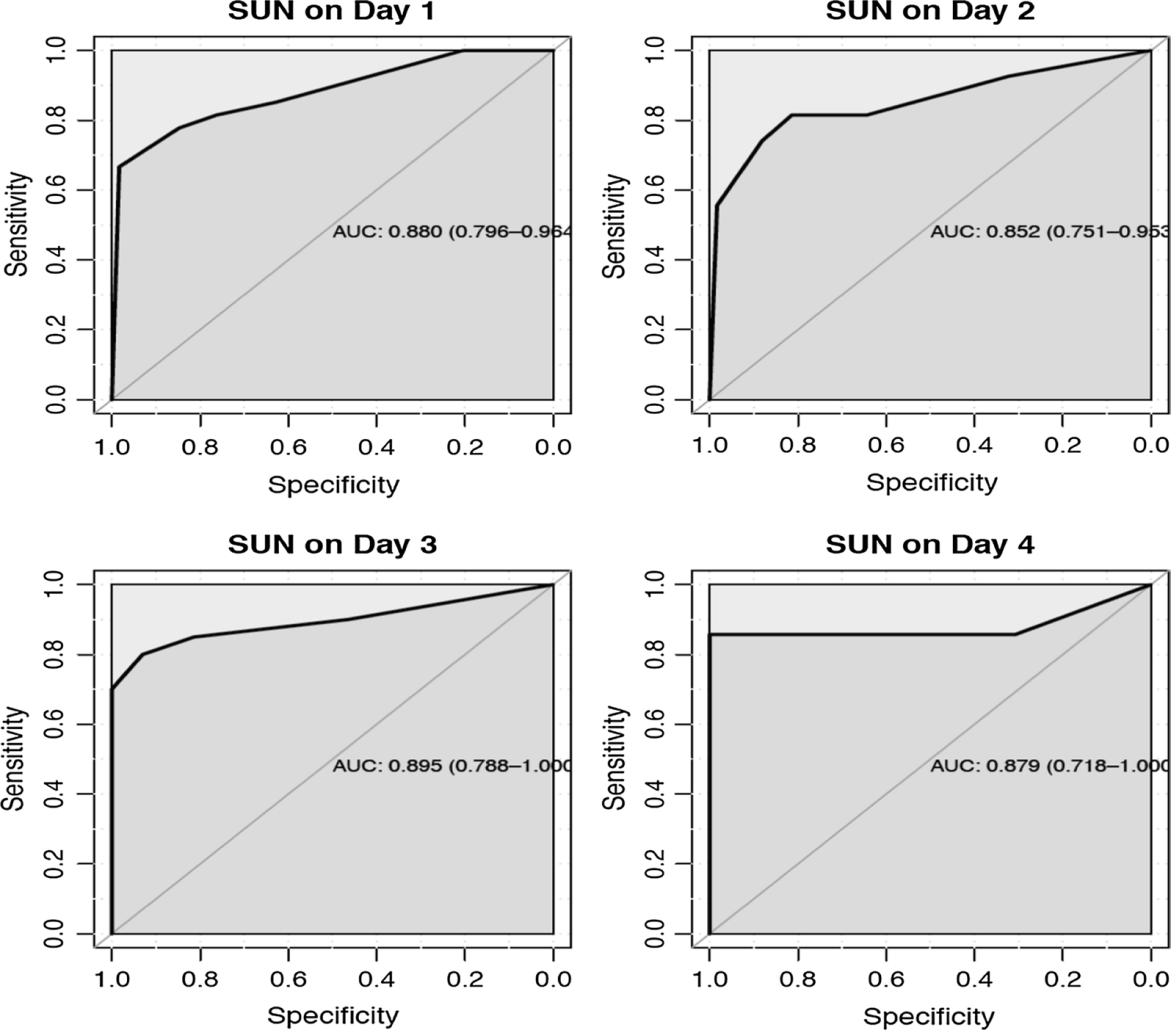
Diagnostic performance of SUN to detect kidney disease at days 1–4. The optimal diagnostic threshold as per Youden Index was SUN test pad #5 (SUN > 54 mg/dL)

For KD diagnosis of, the area under the receiver operator characteristics (ROC) curve for SUN ranged from 0.88 (95% CI 0.79–0.96) on observation day 0 (screening day) to 0.87 (95% CI 0.71–1.0) on observation day 4 (Fig. 4), with the highest sensitivity (0.67) and specificity (0.98) at day 0, respectively. The optimal SUN threshold to diagnose KD in this population was test pad #5 (> 54 mg/dL), with a sensitivity of 67% and specificity of 98%.

## Discussion

### Statement of principal findings

Kidney disease is one of the most serious complications in patients suffering from Malaria and is an independent predictor of death [9]. The severe renal impairment demonstrated in this study is an important finding and may represent the delay of the diagnosis and consequently delay to indicate the adequated treatment for malaria and AKI. The lack of laboratory resources or a bedside diagnostic tool in areas where the disease is highly prevalent can be one of the reasons for underdiagnosed kidney disease and increased mortality rate in this population. In the present study, one-third of patients admitted with malaria had some degree of KD. In these patients, the SUN dipstick showed reasonable sensitivity (67%) and an excellent specificity (98%) to detect KD, both at the moment of diagnosis and also in the follow-up.

### Discussion in the light of other studies

#### Characteristics of the patient population

Patients hospitalized with malaria in the studied setting were younger than in most parts of the studies previously performed testing the SUN strip except for the study performed in Malawi, in which similar mean age was found (301 patients, mean age 25.9 ± 6.45 years). In Brazilian cross sectional in which 44 patients were studied the mean age was 59.5 ± 18 years and in the follow up study performed in Brazil and in the USA, 37 subjects evaluated the mean age was 60 ± 19.1 years old [18, 19]. This finding also corroborates with the literature that shows KD, especially AKI, at the low resources setting is a disease which affects mainly young people and as a consequence of community-acquired disease [12, 20, 27].

#### High incidence of AKD and comparisons to other studies

One-third (27%) of the population analysed in this study presented KD, in contrast to the findings of the previous studies performed in which, KD was defined as those patients with AKI and AKD no AKI. In the adult cohort in Malawi one-fifth (20%) of patients studied had KD and in the obstetric cohort one-sixth (13%) of patients were detected with the disease. These differences may be due to the delay those patients in seeking health care, or due to self-medication strategies that may be common where malaria is endemic. The frequency of malaria-associated AKI raised from 4.7% in 1983 to 1995 to 17.0% in 1996 to 2008 in certain regions [28, 29] which may be related to the reduction of AKI due to other causes (e.g. diarrhoea-associated AKI), rather than changes in virulence of the protozoa.

#### Performance of SUN at the baseline and follow-up

For the diagnosis of KD, the area under the receiver operator characteristics curve for SUN ranged from 0.88 (95% CI 0.79–0.96) on observation Day 0 (screening day) to 0.87 (95% CI 0.71–1.0) on observation day 4 (Fig. 4), with the highest sensitivity (0.67) and specificity (0.98) at day 0, respectively. Similar findings were reported in our previous results with SUN demonstrating a adequated diagnostic performance with similar area under ROC (AUROC) especially at the admission, Malawi adults cohort with 0.82 (95% CI 0.77–0.85) and the Brazilian adult AKI cohort with an AUROC of 0.81 (CI 95% 0.63–0.98) at day 0 [19, 20].

#### Reasons for the better results in comparison to the previous studies

The optimal threshold of SUN to detect kidney impairment in the studied population was considerably higher (test-pad #5) as compared to other studies in AKI patients reported [18, 20]. A possible reason for that is explained by the higher sCr, BUN and SUN values at admission compared to other populations analysed in previous studies and the well described increased diagnostic performance of SUN strips at the higher levels of BUN. The severe renal impairment demonstrated in this group of patients is possibly due to the late admission, prolonged self-management without seeking medical attention, and long travel times [16, 30].

### Study limitations

There are some limitations to the use of SUN dipsticks in general: (a) some bacteria in the oral cavity produce urease that may pre-analytically lower SUN levels and result in a spurious underestimation of BUN [31]; (b) patients with malaria are often dehydrated, resulting in BUN and SUN levels disproportionally increased compared to the kidney function; these circumstances may result in false positive KD diagnoses [10, 32]; (c) poor agreement between SUN and BUN at lower levels is evident in the current data and is consistent with the previous work. While the exact reasons for this finding are still uncertain, it indicates a need for further improvement of the test pads to increase the sensitivity to detect milder (i.e. earlier) stages of renal disease [18–20]. This study may further be limited in terms of generalizability which may be affected by the inclusion of patients at ages between 11 and 50 years. Also the results regarding the mean age of the affected group could have change if older than 50 years old subjects have been included in the study. While the risk of selection bias exists, the intention was to restrict the study to those that are most likely affected by malaria and whose kidney disease is essentially caused by malaria and not a reflection of a pre-existent chronic renal impairment, which could occurred if we included older people with previous chronic co-morbidities, such as hypertension and diabetes, or even in smaller children with acute episodes of renal impairment due to other infectious diseases.

### Strength, interpretation and clinical implications

This is the first study that assessed the diagnostic performance of SUN dipsticks to KD (AKI + AKD no AKI) detection in malaria patients. A satisfactory diagnostic accuracy was found which may favour the dipstick as to be considered a useful diagnostic tool to detect KD in this population. This could be enormously helpful and facilitate early diagnosis and treatment and increase the chances of renal recovery. In developing countries, the SUN dipstick could assist health care workers to diagnose AKI and AKD without AKI and facilitate triage and referral to secondary and tertiary hospitals. To what extent this will translate into an improved survival remains to be explored in future studies. Further developments may render the dipstick an important diagnostic tool for acute nephrology care in developing countries.

## Conclusion

In this cohort, the SUN dipstick identified malaria patients with severe renal impairment with good diagnostic accuracy. SUN identified patients with severe renal impairment with sensitivity and specificity at acceptable levels for clinical care, rendering the dipstick useful in settings with limited resources. In line the previous work, this study corroborates SUN as a potentially helpful tool for the detection of acute renal disease in patients with malaria.

## Additional file

**Additional file 1: Figure S1.** Salivary urea nitrogen dipstick labelling.
