# Supplementary figures and images for: Diagnostic performance of salivary urea nitrogen dipstick to detect and monitor acute kidney disease in patients with malaria
Source: Malar J. 2018 Dec 18;17:477. doi: 10.1186/s12936-018-2627-4 (PMC6299494; doi:10.1186/s12936-018-2627-4)

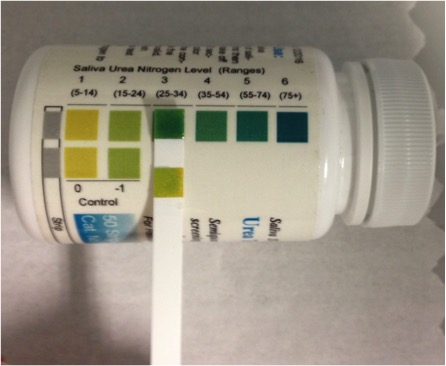

Supplement: Supplementary file 1 — Additional file 1: Figure S1. Salivary urea nitrogen dipstick labelling. [file 12936_2018_2627_MOESM1_ESM.jpg]
